# Artificial Nourishment of Infants

**Published:** 1881-08

**Authors:** 


### Artificial Nourishment of Infants.

In this contribution to the study of the artificial nourishment of infants, Dr. Monti reports a series of observations upon the use of Biedert's artificial food (cream mixture). He says that even diluted cow's milk contains so much casein as to make it hard for the child to digest; further, that a certain amount of fat is essential to the digestion of bovine casein, and in diluting the milk the proper proportion between the casin and the fat is disturbed. Biedert's artificial food consists of white of egg, sugar of milk, cane sugar, butter and salts. Of this one teaspoonful is dissolved in sixteen teaspoonsful of warm water and given to the infant, cow's milk being added by the teaspoonful as the child grows, the amount of it to be added being determined by the child's ability to digest it. The author has tried this mixture in thirty-three cases, and under the following conditions: 1.

As the exclusive food of new-born infants, to test its quality as nourishment. 2. In cases were infants have been artificially nourished, and have lost flesh on account of intestinal troubles, to test its nourishing quality in sick children. 3. As a dietetic means to overcome some intestinal trouble. 4. In children nursed at the breast, but not thriving, in which case it was administered with the breast milk. 5. Given to recently weaned children, who did not readily digest cow's mil. The following results were reached: In the first series of five cases, an increase of body weight was found in all, but in two infants it was not well borne. In the second series, of six cases, all the children were under three months old, with acute and chronic intestinal troubles and emaciation. Within from six to seventeen days after administering the mixture all the intestinal troubles were overcome, and the children gained in weight; but on continuing the mixture for some months together, in two of the cases slight enteritis occurred, but did not end fatally. In the third series, of fifteen children, eleven were less than four months old, and of the whole number thirteen recovered and two died. In the fourth series, of three cases, the results were most satisfactory, the children gaining in weight, though in two cases transitory dyspepsias were observed. In the fifth series, of four cases, all showed the best results.

